# Predictors of temporary epicardial pacing wires use after valve surgery

**DOI:** 10.1186/1749-8090-9-33

**Published:** 2014-02-12

**Authors:** Nizar R AlWaqfi, Khaled S Ibrahim, Yousef S Khader, Ahmad Abu Baker

**Affiliations:** 1 Department of General Surgery, Jordan University of Science and Technology and King Abdullah University Hospital, Princess Muna Heart Center, Floor 8 C, Po Box 630001, Irbid 22110, Jordan; 2 Department of Public Health, Community Medicine, and Family Medicine, Jordan University of Science and Technology and King Abdullah University Hospital, Po Box 630001, Irbid 22110, Jordan; 3 Princess Muna Heart Center, Department of Anesthesia, Jordan University of Science and Technology and King Abdullah University Hospital, Po Box 630001, Irbid 22110, Jordan

**Keywords:** Pacing heart, Temporary pacing, Heart valves

## Abstract

**Background:**

Although temporary cardiac pacing is infrequently needed, temporary epicardial pacing wires are routinely inserted after valve surgery. As they are associated with infrequent, but life threatening complications, and the decreased need for postoperative pacing in a group of low risk patients; this study aims to identify the predictors of temporary cardiac pacing after valve surgery.

**Methods:**

A retrospective analysis of data collected prospectively on 400 consecutive valve surgery patients between May 2002 and December 2012 was performed. Patients were grouped according to avoidance or insertion of temporary pacing wires, and were further subdivided according to temporary cardiac pacing need. Multiple logistic regression was used to determine the predictors of temporary cardiac pacing.

**Results:**

170 (42.5%) patients did not have insertion of temporary pacing wires and none of them needed temporary pacing. 230 (57.5%) patients had insertion of temporary pacing wires and among these, only 55 (23.9%) required temporary pacing who were compared with the remaining 175 (76.1%) patients in the main analysis. The determinants of temporary cardiac pacing (adjusted odds ratios; 95% confidence interval) were as follows: increased age (1.1; 1.1, 1.3, p = 0.002), New York Heart Association class III- IV (5.6; 1.6, 20.2, p = 0.008) , pulmonary artery pressure ≥ 50 mmHg (22.0; 3.4, 142.7, p = 0.01), digoxin use (8.0; 1.3, 48.8, p = 0.024), multiple valve surgery (13.5; 1.5, 124.0, p = 0.021), aorta cross clamp time ≥ 60 minutes (7.8; 1.6, 37.2, p = 0.010), and valve annulus calcification (7.9; 2.0, 31.7, p = 0.003).

**Conclusion:**

Although limited by sample size, the present results suggest that routine use of temporary epicardial pacing wires after valve surgery is only necessary for high risk patients. Preoperative identification and aggressive management of predictors of temporary cardiac pacing and the possible modulation of intraoperative techniques can decrease the need of temporary cardiac pacing. Prospective randomized controlled studies on a larger number of patients are necessary to draw solid conclusions regarding the selective use of temporary epicardial pacing wires in valve surgery.

## Background

Starting 1960s, temporary epicardial pacing wires (PWs) were routinely placed in all cardiac operations for therapeutic as well as diagnostic purposes [1,2]. They remain in place anywhere from 24 hours to several days postoperatively and are used to maintain heart rate and rhythm which are necessary to optimize haemodynamics [3] and to suppress both atrial and ventricular tachyarrhythmias [4]. The use of PWs is associated with increased resource utilization and infrequent, but life threatening complications including hemorrhage, cardiac tamponade, serious arrhythmias, and death. Also the retention of PWs after cardiac surgery is not necessarily safe and may cause serious complications [5-11].

The available data supports the use of PWs for selected cases after coronary artery bypass grafting (CABG) surgery [5,6]. However, PWs are routinely inserted after valve and congenital heart surgeries promoted by historical evidence of increased need for temporary cardiac pacing (TCP) in such surgeries [12-14]. Recent limited publications suggest a trend towards selective use [15,16]. Ferrari et al. [15] reported TCP in 17% of their valve surgery cases and Gupta et al. [16] in 30% of their congenital heart surgery cases.

The aim of this study was to determine the predictors of TCP in valve surgery with the potential to regulate PWs insertion at our center.

## Methods

### Patients

This study is a retrospective review of the clinical, operative, and outcome data that is part of the prospectively recorded cardiac intensive care unit (CICU) database. We excluded patients who underwent redo valve surgery due to their small number and patients with preoperative high degree atrioventricular block (AVB). A total of 400 patients, 18 years or older underwent valve surgery at King Abdullah University Hospital (KAUH), Jordan between May, 2002 and December, 2012, was included in the analysis.

Lately, it is our clinical practice, and upon surgeon’s discretion based on patient’s individual characteristics to selectively insert PWs in valve surgery patients.

According to insertion or avoidance of PWs, two groups of patients were identified: group 1; did not have PWs inserted and never needed to be paced and group 2; had PWs inserted. Group 2 was subdivided into patients who needed TCP and those who did not need TCP. This study was approved by the ethical committee at KAUH.

### Variables

The need for postoperative TCP (if patients were paced at the time of chest closure or at any time before hospital discharge) was the outcome variable of interest. We identified the demographic, clinical, preoperative (including drugs directly affecting the conduction system), and intraoperative variables (including type of valve surgery) as potential predictors of TCP (Table 1). Preoperative arrhythmias were defined as atrial fibrillation (AF), low grade AVB, or a bundle branch block diagnosed by electrocardiogram (ECG). Type of valve surgery was subdivided into; mitral valve, aortic valve, multiple valve (double or triple), and valve with CABG surgeries. There were no cases of only-tricuspid valve surgery, but part of a combined procedure.

**Table 1:** **Characteristics of total patients and by group according to demographic, preoperative, and intraoperative data**^**a**^

**Variable**	**Total**	**Group 1**	**Group 2**	**p-value**
	**(n = 400)**	**(n = 170)**	**(n = 230)**	
Age (year)				< 0.005
< 40	78 (19.5)	62 (79.5)	16 (20.5)	
40 – 59	187 (46.8)	88 (47.1)	99 (52.9)	
≥ 60	135 (33.7)	20 (14.8)	115 (85.2)	
Sex				0.141
Female	177 (44.3)	68 (38.4)	109 (61.6)	
Male	223 (55.7)	102 (45.7)	121 (54.3)	
Renal failure (Creatinine ≥ 1.5 mg/dl)	29 (7.3)	8 (27.6)	21 (72.4)	0.092
Diabetes mellitus	80 (20)	19 (23.7)	61 (76.3)	< 0.005
COPD	49 (12.3)	29 (59.2)	20 (40.8)	0.012
History of arrhythmia	43 (10.8)	3 (7)	40 (93)	< 0.005
NYHA Class				0.027
I – II	280 (70)	129 (46.1)	151 (53.9)	
III – IV	120 (30)	41 (34.2)	79 (65.8)	
Left ventricle ejection fraction (%)				0.006
> 35	259 (64.5)	123 (47.5)	136 (52.5)	
≤ 35	141 (35.5)	47 (33.3)	94 (66.7)	
PAP (mmHg)				0.043
< 50	360 (90)	159 (44.2)	201 (55.8)	
≥ 50	40 (10)	11 (27.5)	29 (72.5)	
Preoperative medications				< 0.005
Beta blockers	107 (26.8)	45 (42.1)	62 (57.9)	
Digoxin	42 (10.5)	19 (55.2)	23 (54.8)	
Amiodarone	18 (4.5)	2 (11.1)	16 (88.9)	
Left atrium diameter (mm)				0.876
< 5.2	347 (86.7)	148 (42.7)	199 (57.3)	
≥ 5.2	53 (13.3)	22 (41.5)	31 (58.5)	
Type of surgery				< 0.005
Mitral valve	140 (35)	80 (57.1)	60 (42.9)	
Aortic valve	129(32.3)	57 (44.2)	72 (55.8)	
Multiple valve	73 (18.2)	21 (28.8)	52 (71.2)	
Valve with coronary artery bypass	58 (14.5)	12 (20.7)	46 (79.3)	
Valve annulus calcification	70 (17.5)	17 (24.3)	53 (75.7)	< 0.005
Cardiopulmonary bypass time (minute)				< 0.005
< 100	317 (79.3)	157 (49.5)	160 (50.5)	
≥ 100	83 (20.7)	13 (15.7)	70 (84.3)	
Aorta cross clamp time (minute)				< 0.005
< 60	318 (79.5)	160 (50.3)	158 (49.7)	
≥ 60	82 (20.5)	10 (12.2)	72 (87.8)	
Intraoperative bradycardia	101 (25.3)	46 (45.5)	55 (54.5)	0.474

^a^ Data are given as no. (%).

COPD = chronic obstructive pulmonary disease; NYHA = New York Heart Association; PAP = pulmonary artery pressure; PW = temporary epicardial pacing wires.

### Operative technique

All patients had median sternotomy. In mitral valve surgery, cardiopulmonary bypass (CPB) was established between the ascending aorta and bicaval venous cannulation. Mitral valve was approached through a conventional left lateral atriotomy. Perfusion was maintained at 2.0 to 2.4 L/min/m^2^, and systemic perfusion pressure was kept at 60 - 80 mmHg. Myocardial protection was achieved by hypokalemic cold crystalloid antegrade cardioplegia (10-12 ml/kg) at a rate of 200 – 250 ml/min, in addition to 300 ml repeated every 10 to 15 minutes. In aortic valve surgery an extra half dose of cardioplegia was given directly into the coronary ostia. Patients were cooled down to 28-32°C. Cold saline was used to cool surface of the heart. Valve annulus debridement of calcium was done usually in a blunt fashion. Patients were evaluated on an individual basis to determine if TCP is required. Patients who received PWs had ventricular wires on the anterior surface of the right ventricle. Atrial wires were additionally placed when AVB occurred after separation from CPB. After surgery all patients were transferred to CICU on mechanical ventilation. Continuous ECG monitoring was used in all patients. 12-lead ECG was performed for all patients upon CICU admission and daily till discharge from hospital.

### Statistical analysis

Statistical analysis was carried out using Statistical Package for Social Sciences (SPSS, version 15). Descriptive statistics were obtained, such as mean values for continuous variables and proportions for categorical variables. The relationships between pacing and possible predictors were analyzed using Chi square test. Multiple logistic regression was used to determine the predictors of pacing in the multivariate analysis. Only factors that were shown to be significant predictors remained in the final regression model. A P-value ≤ 0.05 was considered statistically significant.

## Results

A total of 400 consecutive patients underwent valve surgery with ages ranging from 18 to 80 years with mean age (standard deviation- SD) of 51.81 (13.71) years. Group 1 consisted of 170 (42.5%) patients with mean age (SD) of 44.25 (12.85) years. No patient in group1 required pacing by any means nor suffered any complication attributed to avoidance of PWs. Group 2 consisted of 230 (57.5%) patients with mean age (SD) of 57.39 (11.49) years. Table 1 shows the characteristics of both groups with p-values. Among patients in group 2, only 55 (23.9%) needed TCP (mean age (SD) = 62.27 (10.87) years) and were compared with the remaining 175 (76.1%) patients (mean age (SD) = 55.86 (11.27) years) who did not need TCP. 12-lead ECG was used to diagnose conduction disturbances. The primary reasons for pacing included AVB of any degree in 25 (45.5%) patients, nodal or junctional rhythms in 8 (14.6%) patients, low cardiac output in 13 (23.6%) patients, sinus bradycardia in 5 (9.1%) patients, and asystole in 4 (7.2%) patients. In no case were PWs used for diagnostic purposes, rapid atrial pacing, or left ventricular pacing. PWs were left in place for a minimum of 3 days. The mean (SD) duration of pacing was 53.12 (31.81) hours; the median was 12.5 hours. Only 5 (9.1%) of the 55 patients who needed TCP, had implantation of permanent pacemaker, accounting for 1.25% of the cohort analyzed. The average time from surgery to implantation of permanent pacemaker was 65.33 hours.

Table 2 shows the univariate analysis of demographic, clinical, and preoperative characteristics of patients in group 2, in relation to TCP. The use of TCP differed significantly according to patients’ age, diabetes mellitus, left ventricular ejection fraction (EF), New York Heart Association (NYHA) status, pulmonary artery pressure (PAP), and being on amiodarone. Patients on beta blockers were significantly less likely to need TCP.

**Table 2:** **Univariate analysis (group 2) for use of temporary pacing according to demographic, and preoperative variables**^**a**^

**Variable**	**Temporary cardiac pacing**		
	**No**	**Yes**	**p-value**
Age (year)			0.013
< 40	13 (81.3)	3 (18.8)	
40–59	84 (84.8)	15 (15.20)	
≥ 60	78 (67.8)	37 (32.2)	
Sex			
Female	79 (72.5)	30 (27.5)	0.223
Male	96 (79.3)	25 (20.7)	
Renal failure (creatinine > 1.5 mg/dl)			0.991
No	159 (76.1)	50 (23.9)	
Yes	16 (76.2)	5 (23.8)	
Diabetes mellitus			0.001
No	119 (70.4)	50 (29.6)	
Yes	56 (91.8)	5 (8.2)	
COPD			0.504
No	161 (76.7)	49 (23.3)	
Yes	14 (70.0)	6 (30.0)	
History of arrhythmia			0.818
No	144 (75.8)	46 (24.2)	
Yes	31 (77.5)	9 (22.5)	
NYHA class			0.000
I-II	136 (90.1)	15 (9.9)	
III-IV	39 (49.4)	40 (50.6)	
Left Ventricle ejection fraction (%)			0.001
> 35	114 (83.8)	22 (16.2)	
≤ 35	61 (64.9)	33 (35.1)	
PAP (mmHg)			0.000
< 50	171 (85.1)	30 (14.9)	
≥ 50	4 (13.8)	25 (86.2)	
Left atrium diameter (mm)			0.104
< 5.2	155 (77.9)	44 (22.1)	
≥ 5.2	20 (64.5)	11 (35.5)	
Beta blockers			0.002
No	119 (70.8)	49 (29.2)	
Yes	56 (90.3)	6 (9.70)	
Digoxin			0.440
No	159 (76.8)	48 (23.2)	
Yes	16 (69.6)	7 (30.4)	
Amiodarone			0.011
No	167 (78.0)	47 (22.0)	
Yes	8 (50.0)	8 (50.0)	

^a^Data are given as no. (%).

COPD = chronic obstructive pulmonary disease; NYHA = New York Heart Association; PAP =pulmonary artery pressure.

Table 3 shows the univariate analysis of intraoperative variables in relation to TCP. When analyzed by type of surgery; 10% of patients who underwent mitral valve, 13.9% who underwent aortic valve, 34.8% who underwent valve with CABG, and 44.2% who underwent multiple valve surgeries needed TCP.

**Table 3:** **Univariate analysis (group 2) for use of temporary pacing according to procedure and intraoperative variables**^**a**^

**Variable**	**Temporary cardiac pacing**		
	**No**	**Yes**	**p-value**
Type of surgery			0.000
Mitral valve	54 (90.0)	6 (10.0)	
Aortic valve	62 (86.1)	10 (13.9)	
Multiple valve	29 (55.8)	23 (44.2)	
Valve with coronary artery bypass	30 (65.2)	16 (34.8)	
Annulus Calcification			0.000
No	154 (87.0)	23 (13.0)	
Yes	21 (39.6)	32 (60.4)	
Cardiopulmonary bypass time (minute)			0.000
< 100	140 (87.5)	20 (12.5)	
≥ 100	35 (50.0)	35 (50.0)	
Aorta cross clamp time (minute)			0.000
< 60	143 (90.5)	15 (9.5)	
≥ 60	32 (44.4)	40 (55.6)	
Bradycardia			0.003
No	125 (71.4)	50 (28.6)	
Yes	50 (90.9)	5 (9.1)	

^a^Data are given as no. (%).

Table 4 shows the predictors of TCP in the multivariate analysis. Increased age by 1 year was associated with increased odds of TCP by 10%. Those who underwent multiple valve surgery were significantly more likely to need TCP compared to patients who underwent single valve surgery.

**Table 4:** Multivariate analysis of factors associated with temporary cardiac pacing

**Variable**	**Odds ratio (OR)**	**95% confidence interval (CI)**	**P-value**
Age	1.1	(1.1, 1.3)	0.002
NYHA class (III- IV)	5.6	(1.6, 20.2)	0.008
PAP ≥ 50 mmHg	22.0	(3.4, 142.7)	0.001
Preoperative digoxin use	8.0	(1.3, 48.8)	0.024
Type of surgery			
Mitral valve	1.0		
Aortic valve	2.2	(0.3, 14.7)	0.431
Multiple valve	13.5	(1.5, 124.0)	0.021
Valve with coronary artery bypass	3.6	(0.4, 30.7)	0.242
Aorta cross clamp time ≥ 60 min	7.8	(1.6, 37.2)	0.010
Valve annulus calcification	7.9	(2.0, 31.7)	0.003

NYHA = New York Heart Association; PAP = pulmonary artery pressure.

## Discussion

Historically, PWs have been routinely used after cardiac operations to optimize cardiac function and to suppress both atrial and ventricular tachyarrhythmias [4]. However, their use is not innocuous and associated with infrequent but lethal complications. Insertion can prolong the operative time and increase the risk of bleeding. Their extended use can result in failure of sensing or capturing [17]. During removal, patients are at risk of ventricular arrhythmias [18] and to a lesser extent bleeding due to injury of nearby structures [19-21]. Also retention of PWs may be complicated by deep seated infections or migration to different structures in the chest [7-9,22,23].

During the last decade, routine insertion of PWs in CABG surgery, being on-pump or a beating heart, has been well studied and many centers turned to limit their use [5,6,24]. We already comply with these policies. In valvular and pediatric surgeries there is a historical evidence of routine use of PWs due to the increased risk of AVB [12]. However, these recommendations are decades old and may not reflect modern surgical techniques and practice. Recent few reports studied the rate and the predictors of TCP after valvular and pediatric cardiac surgeries [15,16,25]. At our center, to avoid the above mentioned complications and in accordance with the published literature, and it is upon surgeon’s discretion to safely select patients, we limited the insertion of PWs in valve surgery (usually based on; young age with minimal comorbidities, uncomplicated single valve surgery, not involving extensive decalcification, and after separation from CPB in sinus rhythm, and hemodynamically stable on minimal support, including recently, patients in sinus bradycardia who responds to minimal doses of beta-adrenergic drugs) (Table 1).

The need for TCP was mainly based on the presence of a mechanical injury to the conduction system caused by operative procedures in close physical proximity to the atrioventricular node or the His bundle, or an ischemic injury to the conduction system resulting during cardioplegic arrest, especially if associated with extensive coronary artery disease. Both mechanisms may evoke preexisting conduction defects or generate new ones.

In our study, only 23.9% of valve surgery patients with PWs insertion needed TCP which led us to reevaluate the use of PWs and to attempt to identify a subpopulation of valve patients for whom PWs are appropriate.

In the current series, patients who had PWs inserted were almost 10 years older than those who did not, and those more than 60 years old were, by both univariate and multivariate analysis, more likely to be paced.

Gender was not a predictor of TCP. This is consistent with other peer reviews [15,26].

Data in the literature regarding the significance of chronic comorbidities such as chronic renal failure, chronic obstructive pulmonary disease, diabetes mellitus and preoperative arrhythmias as predictors of postoperative pacing are conflicting [14,15,27]. In our study none of the above mentioned comorbidities were significantly associated with the need for TCP in the multivariate analysis.

Advanced NYHA class was a predictor of TCP in both univariate and multivariate analysis. Pacing was used in patients with advanced NYHA class to improve cardiac output after separation from CPB to achieve hemodynamic stability. Also, most of them had severe left ventricular dysfunction with increased risk of developing various conduction disturbances [28]. This finding was supported by Gordon et al. [29]. On the contrary, advanced NYHA status was not a predictor of TCP by Ferrari et al. [15].

Increased PAP was an independent predictor of TCP by both univariate and multivariate analysis. Limongelli, et al. [30] reported pulmonary hypertension a risk factor for postoperative AVB following aortic valve surgery, as it acts on the right ventricular dimensions and shape, and interventricular septal thickness imposing progressive mechanical stretch that could affect the conduction system by altering the electrophysiological properties of its fibers.

Preoperative use of digoxin and beta-blockers are postulated as predictors of postoperative conduction disturbance [14,31], especially of those with the block nature. In a recent study on patients after mitral valve surgery by Berdjas et al. [14], both digoxin and beta blockers, were found to significantly lower the risk of conduction disturbances. In our study, only digoxin was a predictor of TCP. Digoxin’s primary mechanism of action is to increase the force of myocardial contractility in failing hearts by increasing the intracellular calcium concentration. However, its vagomimetic effect decreases sinoatrial and atrioventricular conduction and prolongs atrioventricular node refractory period, thus increasing the need for TCP [32]. Also digoxin has, even at therapeutic levels, an arrhythmogenic effect on ischemic hearts [33], which may include periods of prolonged crossclamping time.

Type of surgical procedure was a determinant of TCP. Patients who had multiple valve surgery and/or debridement of heavy annular calcification at the atrioventricular sinus area (the posterior commissure of the anterior mitral leaflet, the commissure between right- and noncoronary cusps of the aortic valve, and the septal leaflet of the tricuspid valve) were at an increased risk of physical injury to the atrioventricular node and conduction system and were significantly more likely to need TCP after separation from CPB. Although the need for pacing in combined valve and CABG surgery was more than in isolated single valve surgery, this difference did not reach statistical significance. This might be due to small sample size of this subgroup. In such patients it may be difficult to assure adequate and uniform delivery of the cardioplegic solution to protect the myocardium leading to new or further exacerbating existing conduction disturbances. These results coincide with other peer reviews [29,34].

Aortic crossclamp time more than 60 minutes, by both univariate and multivariate analysis, was a predictor of TCP. This is consistent with other peer reviews [35]. The occurrence of ischemic injury to the conduction system and the development of myocardial edema following prolonged periods of crossclamping might explain these findings.

In the interpretation of the study findings, we need to consider that this study is a retrospective analysis with a relatively limited number of patients done over a long time; however patients were followed prospectively during their hospitalization. Although a team of different surgeons performed the operations with different threshold for postoperative PWs insertion and pacing, all the patients were treated at a single center, and the operative methods remained substantially unchanged during the study.

## Conclusion

Although limited by sample size, the present results suggest that routine use of PWs after valve surgery is necessary for high risk patients. Predictive factors of TCP after valve surgery were increased age, NYHA class (III-IV), PAP ≥ 50 mmHg, digoxin use, complex surgical procedures (i.e. multiple valve surgery), heavy annular calcification, and crossclamp time ≥ 60 min. Better patients selection, in addition to optimizing the medical condition through aggressive treatment of heart failure and pulmonary hypertension, shortening the cross clamping and CPB times, and careful debridement of calcified valve annulus, may lower the need for postoperative pacing in our practice. Prospective randomized well controlled studies on a larger number of patients, including quantitative assessment of valve annular calcium load and distribution, are necessary to draw solid conclusions on safely selected patients who do not require postoperative PWs insertion.

## Abbreviations

PWs: Temporary epicardial pacing wires; CABG: Coronary artery bypass grafting; TCP: Temporary cardiac pacing; CICU: Cardiac intensive care unit; KAUH: King Abdullah University Hospital; AVB: Atrioventricular block; AF: Atrial fibrillation; ECG: Electrocardiogram; CPB: Cardiopulmonary bypass; SD: Standard deviation; EF: Left ventricular ejection fraction; NYHA: New York Heart Association; PAP: Pulmonary artery pressure.

## Competing interests

The authors declare that they have neither financial nor non-financial competing interests.

## Authors’ contribution

NA participated in clinical practice, contributed to conception, design, acquisition of data, drafting the manuscript, and revising it critically for important intellectual content. KI participated in clinical practice, contributed to design, helped in drafting the manuscript, and revising it critically for important intellectual content. YK contributed to design, analysis and interpretation of data, and helped in drafting the manuscript. AA participated in clinical practice, helped in drafting the manuscript, and revising it critically for important intellectual content. All authors read and approved the final manuscript.
